# A Case of Ertapenem-Induced Delirium

**DOI:** 10.7759/cureus.52732

**Published:** 2024-01-22

**Authors:** Maria Aquilina, Nicole Galdes, Neville Spiteri

**Affiliations:** 1 Surgery, Mater Dei Hospital, Msida, MLT; 2 Colorectal Surgery, Mater Dei Hospital, Msida, MLT

**Keywords:** ertapenem side effect, ertapenem-induced encephelopathy, osteomyelitis with abscess, sacral pressure ulcer, acute delirium

## Abstract

Ertapenem is a carbapenem antibiotic that is typically prescribed in cases of moderate-to-severe infections, especially ones involving abscess formation. We describe the case of an 82-year-old gentleman who presented with osteomyelitis and abscess formation who developed delirium after 15 days of ertapenem treatment. The patient experienced delusions, insomnia, agitation, and disorientation. The patient’s mental status improved and returned to his baseline within 48 hours of halting ertapenem treatment. A high index of suspicion is required to identify and treat ertapenem-induced delirium. Withdrawal of ertapenem treatment in such cases usually results in a complete resolution of symptoms.

## Introduction

Ertapenem is a member of the carbapenem class of antibiotics [1,2]. It is a broad-spectrum antibiotic and is administered via the intravenous route [1,2]. Resistance to ertapenem is uncommon in species that are susceptible to this treatment [2]. It is also usually effective in the treatment of Enterobacteriaceae with extended-spectrum beta-lactamases (ESBLs) [2]. It is typically prescribed in cases of moderate-to-severe infections, especially ones involving abscess formation.

Here, we describe the case of an 82-year-old gentleman who presented with osteomyelitis and abscess formation who experienced delirium secondary to ertapenem treatment.

## Case presentation

Our patient is an 82-year-old gentleman who was admitted from the community in view of sepsis from an infected sacral sore.

He has a known case of polio affecting both his lower limbs. He is bedridden and has a long-term urinary catheter in situ. He presented with two pressure sores, one over the sacrum and a second one over the left hip. Computed tomography (CT) taken on admission confirmed chronic osteomyelitis with abscess formation. He was immediately started on intravenous piperacillin/tazobactam (tazocin) 4.5 g eight-hourly and intravenous teicoplanin 600 mg 12-hourly for four doses, to continue at a dose of 600 mg once-daily thereafter.

Debridement of the ulcers was performed on day two of admission. Tissue taken during debridement cultured the following bacteria: *Morganella morganii*, *Pseudomonas aeruginosa, *and *Staphylococcus aureus*. The full sensitivity results can be found in Table 1. In view of these results, piperacillin/tazobactam was increased to 4.5 g six-hourly to target the intermediate-sensitivity *Pseudomonas aeruginosa*. In addition, teicoplanin was stopped after 10 days of treatment and intravenous clindamycin 600 mg eight-hourly was started to address the growth of *Staphylococcus aureus*.

**Table 1:** Culture and sensitivity results from tissue taken during the debridement of the ulcers on day two of admission with the choice of antibiotics in bold font R: resistant, I: intermediate sensitivity, S: sensitive

Antibiotic	Sensitivities		
	Morganella morganii	Pseudomonas aeruginosa	Staphylococcus aureus
Penicillin			R
Co-amoxiclav	R		
Piperacillin/tazobactam	S	I	
Ceftazidime		I	
Imipenem	I	I	
Amikacin		S	
Oxacillin/flucloxacillin			S
Levofloxacin			I
Clindamycin			S
Gentamicin	S		
Tobramycin		S	
Ciprofloxacin	R	I	
Colistin		S	
Tetracycline			S
Trimethoprim/sulfamethoxazole	R		S

**Figure 1:**
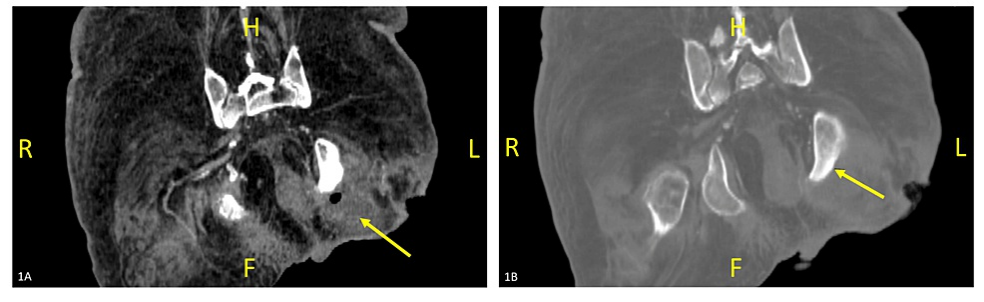
Coronal non-contact CT scan of the pelvis taken on day 20 of admission: 1A: Snapshot with arrow indicating fluid collection with gas adjacent to the left ischial bone. 1B: Snapshot with arrow indicating cortical loss with osteolysis of the left ischial bone, suggestive of osteomyelitis H: head, F: foot, R: right, L: left

Repeat CT scan on day 20 of admission showed persistence of the collection and osteomyelitic changes, as shown in Figure 1A and Figure 1B. Hence, open drainage of the abscess was performed on day 22 of admission. A drain was left in situ for eight days and removed after the wound stopped discharging. Pus that was sampled during this procedure cultured the following bacteria: *Klebsiella pneumoniae*, *Morganella morganii,* and *Clostridium cadaveris*. Full sensitivity results are listed in Table 2. Piperacillin/tazobactam was switched to intravenous ertapenem 1 g once-daily on day 25 of admission. This choice factored in the culture and sensitivity results (Table 2), the requirement for a prolonged course of antibiotic treatment for osteomyelitis, and improved patient comfort due to a reduced dosing frequency. Clindamycin was continued to target *Clostridium cadaveris* although this was switched to oral clindamycin 300 mg six-hourly.

**Table 2:** Culture and sensitivity results from pus sampled during incision and drainage of the sacral abscess on day 22 of admission with the updated choice of antibiotics in bold font R: resistant, I: intermediate sensitivity, S: sensitive

Antibiotic	Sensitivities		
	Klebsiella pneumoniae	Morganella morganii	Clostridium cadaveris
Amoxicillin	R	R	S
Penicillin			S
Co-amoxiclav	R	R	S
Piperacillin/tazobactam	R	S	
Ceftazidime	R		
Ertapenem	S	S	
Clindamycin			S
Metronidazole			S
Meropenem	S	S	
Gentamicin	S	S	
Ciprofloxacin	R	R	
Trimethoprim/sulfamethoxazole	S	R	

On day 39 of admission/day 15 of ertapenem treatment, the patient was noticed to be acutely confused. He was experiencing acute delusions, insomnia, and agitation and was not oriented to time, place, or person.

**Figure 2:**
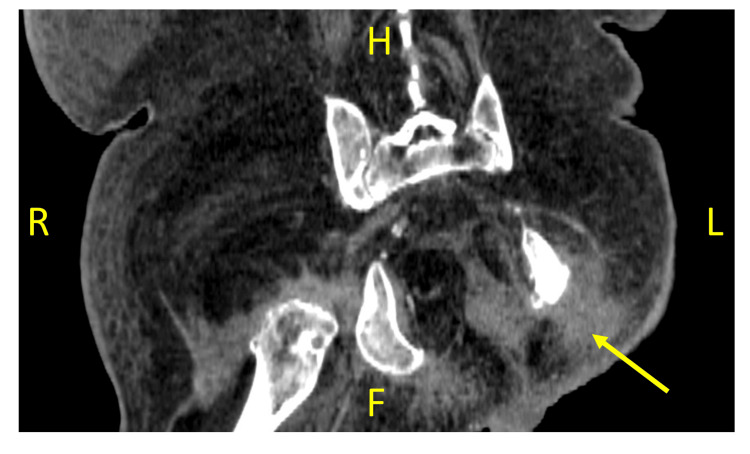
Snapshot from a coronal non-contrast CT scan of the pelvis taken on day 37 of admission. Arrow indicates the site of the previous fluid collection in the left buttock adjacent to the left ischium, which was drained on day 22 of admission H: head, F: foot, R: right, L: left

Physical examination revealed no new changes. The known pressure sores were healing well, and repeat CT performed two days prior to this episode on day 37 of admission showed resolution of the fluid collection, as shown in Figure 2. There was no clear focus of infection or acute neurological deficit aside from the acute confusion. There was no evidence of cardiovascular, respiratory, or abdominal pathology. His parameters remained stable, and blood tests were at his baseline. He had normal liver and renal function. There were no metabolic or electrolyte abnormalities that could explain this acute deterioration. ECG demonstrated sinus rhythm, chest X-ray was unremarkable, and a CT scan of the brain showed no acute intracranial pathology. Urinalysis and urine culture were unremarkable.

Given all these findings, the patient’s treatment was reviewed, and ertapenem was identified as a potential cause of delirium. Hence, this was stopped, and the patient was switched back to piperacillin/tazobactam 4.5 g six-hourly.

The patient’s mental status improved and returned to his baseline within 48 hours of halting ertapenem treatment. He was eventually discharged home after completing a 66-day course of intravenous antibiotic treatment for chronic osteomyelitis.

## Discussion

A review of the drug information available in the British National Formulary (BNF) and Summary of Product Characteristics (SPC) for ertapenem indicates that confusion and insomnia are uncommon side effects of the treatment (≥1/1,000 to <1/100) [1,2]. In addition, the SPC lists the incidence of agitation as rare (≥1/10,000 to <1/1,000) and altered mental status, including aggression, delirium, and disorientation, as frequency is not known [2].

A pharmacovigilance study of the FDA Adverse Event Reporting System (FAERS) revealed that ertapenem had the strongest statistically significant reporting odds ratio (ROR) for developing delirium as an adverse effect of treatment when compared to other antibiotics that have been reported to cause delirium [3]. Thus, even though delirium is an uncommon side effect of ertapenem treatment, it is more likely to occur with ertapenem than with other antibiotics.

Review of the literature reveals a number of case reports and case series wherein patients experienced acute delirium or encephalopathy secondary to ertapenem treatment [4-11]. Reported symptoms included hallucinations [4,5,7,9,11], suicidal ideation [11], delirium [4-11], agitation [4,7,10], seizures [8], encephalopathy (neurotoxicity) [4-11], miosis [6], dysphasia [5,6], and altered mental status [4-11]. Common risk factors in these reports were older patients [6-9,11], prolonged treatment for more than seven days [4-7,9-11], and renal impairment [4-6,8,9]. This is mostly consistent with our case as our patient is an elderly gentleman who was receiving a prolonged course of treatment for osteomyelitis with abscess formation. Our patient, however, maintained a normal renal function throughout the duration of his hospital stay, including the period of ertapenem treatment.

Most cases of ertapenem-induced delirium were characterized by quick recovery (24-72 hours) upon cessation of treatment [5,7-11]. This is reflected in the case described since the patient's mental status returned back to baseline within 48 hours of halting ertapenem treatment.

Another common factor in these reports was a delayed recognition of the link between delirium and ertapenem treatment [7,8,10]. This highlights the importance of pharmacovigilance in clinical practice, especially when dealing with multi-morbid patients.

## Conclusions

We describe a case of ertapenem-induced delirium occurring after 15 days of treatment. A high index of suspicion is required to identify and take prompt action in patients presenting with suspected ertapenem-induced delirium.

This can present late after commencing drug therapy. The elderly are more vulnerable to medicine side effects, including less common ones. Withdrawal of ertapenem treatment in such cases usually results in complete resolution of symptoms.
